# The Prevention of Tuberculosis

**Published:** 1908-10-10

**Authors:** 


### Public Health and Hygiene.

### THE PREVENTION OF TUBERCULOSIS.

Dr. Newsholme's prolific pen has not rested with his elevation to the Principal Medical Officership of the Local Government Board. His latest book, the "Prevention of Tuberculosis,"* was for the most part written, he tells us, while he was Medical Officer of Health of Brighton and almost exclusively from the standpoint of the public health administrator. The thesis is one with which Dr. Newsholme is particularly identified, his contributions notably to the Epidemiological Society and to the *Journal of Hygiene* having already developed his view that "institutional segregation is the predominant cause of the decline of phthisis in this country."

The indefatigable industry with which Dr. Newsholme has ransacked the records and statistics of tuberculosis, and the erudite ingenuity with which he has laid them under contribution to his thesis, command admiration; and if we fail to be convinced by his argument, we appreciate the zeal with which the quest has been pursued.

Differing fundamentally as we do from the main contention, we confess our appreciation of the ability, industry, and effort engaged in the collection, examination, and tabulation of so vast an amount of statistical and other material bearing intimately upon the many problems entailed in the control and treatment of tuberculosis.

The book is marred, however, by the insistent continuity of the argument. So many other inferences than those drawn by the author are to be deduced from the wealth of fact which the book embraces. There is a strained effort to sustain the thesis, the impartial and unbiassed inquiry of the scientist is disturbed by the zealous contention of the controversialist, and the book stands to a scientific treatise very much as a "novel with a purpose" does to a work of art. It is polemical, sectarian, doctrinaire, and purblind to the complex issues of the pathological problem attacked. The health administrator and medical reformer have run away with the calm judgment of the physician-naturalist, and the sanitary priest has fallen, not unnaturally, into the priestly error of dogmatism.

"Each group of records shows, not as a matter of hypothesis or theory, but as the teaching of actual experience, which gives the final touchstone for final conclusions and action, that with no more precautions than are taken in well-conducted general infirmaries the increase of institutional segregation has been associated with reduction of tuberculosis in the community affected by it. . . . No influence except that of institutional segregation has appeared in actual experience in a constant relation to the amount of tuberculosis, and it must, therefore, be accepted as having been the predominant influence."

Now susceptibility is a *sine quâ non* of infectibility, and the varying ratios of susceptible material in a population to constantly changing effective doses of infection in the environment are not measureable even by Dr. Newsholme's intricate statistical methods. It is not so much the specific exposure to tubercular infection which the family physician fears for his patient; it is his known vulnerability, his openness to receive it. The tubercle bacillus may not be ubiquitous—though we are not satisfied that this is proved; but a pneumococcal pleurisy—as Dr. Newsholme well recognises—a typhoidal or measles pneumonia, an alcoholic habit, a bad personal or even racial heredity, do not require to be followed by known specific exposures to infection to be followed by tubercular disease. Without denying prolonged latency as a phenomenon of tuberculosis, we think it mere unwarranted assumption to date it from a supposed lethal exposure during the illness of the last family victim of the disease. It is true this method, unsupported by any evidence of causal connection during possibly a 20 years' interval goes far to dispose of observed hereditary family predisposition; but explanations based on such unsupported theories are not convincing.

But to go to the root of the contention, it is, to say the least, highly improbable that the so-called institutional segregation of consumptives has in any appreciable degree immobilised the infection of tubercle. It is admitted that domestic infection in the home of the consumptive is by far the greatest risk, but can it seriously be contended that during the last 50 years this risk has been materially reduced by the degree of segregation in ill-conducted workhouse infirmaries which Dr. Newsholme's figures may be accepted as proving to have occurred? It is not claimed that, except during part of the last stages of the disease, the consumptive has been removed from the family, which, if susceptible, he must already have efficiently infected. And it is essential to Dr. Newsholme's argument that it is the family which alone would benefit prophylactically at this stage of the disease.

Ingenious statistics are too delicate an instrument to carry conviction when they fail to recognise so elementary an objection to their seeming proof. We do not doubt that Dr. Newsholme, by equally correct statistical methods would be able to show on as large a scale of observations that the decline in the amount of tuberculosis has gone on in an equally constant relation with, say, the increase in production of industrial dust. But the increase of industrial dust, which may be correlated to the decline of phthisis, is not causally related—directly at all events; and we think that such institutional segregation as has occurred bears about the same ætiological relation.

It is not so much that Dr. Newsholme cannot see the wood for trees, as that one big tree, ardently desired by the zealous health crusader, obstructs his vision. We think the influence of institutional segregation upon the decline of phthisis to be a chimera to which the whole argument has been sacrificed, but the book, apart from its argument, is a monument to the industry, skill, and erudition of its author.

* "The Prevention of Tuberculosis." By Arthur Newsholme, M.D., F.R.C.S. (Methuen and Co. 10s. 6d. net.)

